# Identification of key genes and pathways involved in T-DM1-resistance in OE-19 esophageal cancer cells through bioinformatics analysis

**DOI:** 10.1016/j.heliyon.2024.e37451

**Published:** 2024-09-06

**Authors:** Fateme Yazdani, Negar Mottaghi-Dastjerdi, Behzad Shahbazi, Khadijeh Ahmadi, Abozar Ghorbani, Mohammad Soltany-Rezaee-Rad, Hamed Montazeri, Farzane Khoshdel, Pietro Hiram Guzzi

**Affiliations:** a Department of Pharmacognosy and Pharmaceutical Biotechnology, School of Pharmacy, Iran University of Medical Sciences, Tehran, Iran; b School of Pharmacy, Semnan University of Medical Sciences, Semnan, Iran; c Infectious and Tropical Diseases Research Center, Hormozgan Health Institute, Hormozgan University of Medical Sciences, Bandar Abbas, Iran; d Nuclear Agriculture Research School, Nuclear Science and Technology Research Institute (NSTRI), Karaj, Iran; e Behestan Innovation Factory, Tehran, Iran; f Department of Surgical and Medical Sciences, University “Magna Græcia” of Catanzaro, Catanzaro, Italy

**Keywords:** Esophageal cancer, T-DM1 resistance, Hub genes, IL-17 signaling pathway, ECM-Receptor interaction, DrugBank, NSAIDs, Bioinformatics

## Abstract

**Introduction:**

Esophageal Cancer (EC) ranks among the most common malignancies worldwide. Most EC patients acquire drug resistance to chemotherapy either intrinsically or acquired after T-DM1 treatment, which shows that increasing or decreasing the expression of particular genes might influence chemotherapeutic sensitivity or resistance. Therefore, gaining a deeper understanding of the altered expression of genes involved in EC drug resistance and developing new therapeutic methods are essential targets for continued advancement in EC therapy.

**Methods:**

The present study aimed to find critical regulatory genes/pathways in the progression of T-DM1 resistance in OE-19 EC cells. Expression datasets were extracted from GEO omnibus. Gene interactions were analyzed, and the protein-protein interaction network was drawn. Then, enrichment analysis of the hub genes and network cluster analysis of the hub genes was performed. Finally, the genes were screened in the DrugBank database as therapeutic targets and molecular docking analysis was done on the selected targets.

**Results:**

In the current study, nine hub genes were identified in TDM-1-resistant EC cells (CTGF, CDH17, THBS1, CXCL8, NRP1, ITGB5, EDN1, FAT1, and PTGS2). The KEGG analysis highlighted the IL-17 signaling pathway and ECM-receptor interaction pathway as the most critical pathways; cluster analysis also showed the significance of these pathways. Therefore, the genes involved in these two pathways, including CXCL8, FSCN1, PTGS2, SERPINE2, LEF1, THBS1, CCN2, TAGLN, CDH11, and ITGA6, were searched in DrugBank as therapeutic targets. The DrugBank analysis suggests a potential role for Nonsteroidal Anti-Inflammatory Drugs (NSAIDs) in reducing T-DM1 drug resistance in EC. The docking results revealed that NSAIDs, including Diclofenac, Mefenamic acid, Celecoxib, Naproxen, and Etoricoxib, significantly suppress resistant cancer cells.

**Conclusion:**

This comprehensive bioinformatics analysis deeply explains the molecular mechanisms governing TDM-1 resistance in EC. The identified hub genes and their associated pathways offer potential targets for therapeutic interventions. Moreover, the possible role of NSAIDs in mitigating T-DM1 resistance presents an intriguing avenue for further investigation. This research contributes significantly to the field and establishes a basis for further research to enhance treatment efficacy for EC patients.

## Glossary

**T-DM1 (Trastuzumab emtansine)**: An antibody-drug conjugate used in cancer treatment
**Esophageal Cancer (EC)**: A type of cancer that affects the esophagus
**HER2 (Human Epidermal Growth Factor Receptor 2)**: A protein that can promote the growth of cancer cells
**ADC (Antibody-Drug Conjugate)**: A targeted cancer therapy combining an antibody with a cytotoxic drug
**ABC Transporters (ATP-Binding Cassette Transporters)**: Proteins that transport various molecules across cellular membranes
**MDR1 (Multidrug Resistance Protein 1)**: A protein that pumps foreign substances out of cells
**PPI (Protein-Protein Interaction) Network**: A network showing how proteins interact with each other within a cell
**GEO (Gene Expression Omnibus)**: A public database for gene expression data
**DEGs (Differentially Expressed Genes)**: Genes that show different expression levels between two or more conditions
**KEGG (Kyoto Encyclopedia of Genes and Genomes)**: A database resource for understanding high-level functions and utilities of the biological system
**GO (Gene Ontology)**: A framework for the model of biology that relates to gene functions
**CytoHubba**: A Cytoscape plugin used to identify and rank important nodes in a network
**STRING (Search Tool for the Retrieval of Interacting Genes/Proteins)**: A database of known and predicted protein-protein interactions
**IL-17 Signaling Pathway**: A pathway involved in inflammation and immune responses
**ECM (Extracellular Matrix)**: A network of proteins and other molecules outside cells that provides structural and biochemical support to surrounding cells
**NSAIDs (Nonsteroidal Anti-Inflammatory Drugs)**: A class of drugs that reduce pain, decrease fever, and, in higher doses, decrease inflammation
**Urokinase Plasminogen Activator (uPA)**: An enzyme involved in the breakdown of blood clots
**Microarray**: A laboratory tool used to detect the expression of thousands of genes at the same time.
**Bioinformatics**: The use of computational tools to manage and analyze biological data
**Enrichment Analysis**: A method used to identify categories of genes or proteins that are over-represented in a large set of genes or proteins
**Hub Genes**: Genes that play a central role in gene regulatory networks
**Volcano Plot**: A type of scatter plot that shows statistical significance versus magnitude of change
**UMAP (Uniform Manifold Approximation and Projection)**: A dimension reduction technique for visualizing high-dimensional data
**Clustering Algorithm**: A method used to group a set of objects in such a way that objects in the same group are more similar to each other than to those in other groups
**Kaplan-Meier Curve**: A statistical method used to estimate the survival function from lifetime data
**Molecular Docking**: A method used to predict the preferred orientation of one molecule to a second when bound to each other
**Ligand**: A molecule that binds to another (usually larger) molecule
**Receptor**: A protein molecule that receives and responds to a signal molecule
**Autodock Vina**: A software used for molecular modeling and docking studies
**CytoCluster**: A Cytoscape plugin used for the clustering of network node T-DM1 (Trastuzumab emtansine): An antibody-drug conjugate used in cancer treatment

## 1 Introduction

Esophageal cancer (EC) is a type of cancer that affects the esophagus and ranks seventh in incidence and sixth in mortality worldwide, with over 604,100 new cases and about 544,076 deaths in 2020 [1].

HER2, or the human epidermal growth factor receptor 2, is a member of the ErbB/HER receptor tyrosine kinase family that plays a role in various cancers' normal development and oncogenesis. HER2 overexpression has been observed in about 20 % of breast cancers and is linked to poor prognosis and high recurrence risk [2]. Gastric and esophageal cancer have a less than 20 % survival rate after five years [1]. About 20 % of gastric cancers (GC) and 33 % of gastroesophageal junction (GEG) cancers have shown HER2 overexpression [3]. Antibody-drug conjugates (ADCs) combine tumor-targeting antibodies with highly potent cytotoxic agents to treat cancer. They aim to increase the efficacy of chemotherapy while reducing side effects. Trastuzumab emtansine (T-DM1) is one of the four ADCS FDA-approved drugs [4]. Trastuzumab was approved in 2010 for treating patients with metastatic gastric or GEJ adenocarcinomas that overexpress HER2 and have not received prior treatment for metastatic disease [5].

T-DM1 targets HER2 with its antibody component because it is an antibody-drug conjugate (ADC) attached to DM1 via a non-cleavable thioether linker [5]. T-DM1 retains the mechanism of action of trastuzumab besides the antimitotic activity of DM1 [6]. DM1, derived from maytansine, is a powerful antimitotic agent. The mechanism of action is to bind to tubulin at the same site as Vinca alkaloids. After the ADC binds to HER2, the release of the active metabolites requires internalization and processing [5]. After lysosomal degradation of T-DM1, lysine-Nε-SMCC-DM1 is the only metabolite that can be quantified [7]. T-DM1 was approved as a second-line therapy in 2013 for patients with HER2-positive metastatic breast cancer [5]. The efficacy of T-DM1 against uterine, bladder, lung, and gastric cancers that overexpress HER2 has been shown both *in vitro* and *in vivo* [[8], [9], [10], [11], [12]]. Currently, its effectiveness is being tested in patients with HER2-positive GC. Because resistance to therapy will be eventually observed in some patients treated with T-DM1 [5], identifying the resistance mechanism to this agent is essential.

One of the main drawbacks of anti-cancer drugs is the development of resistance to treatment [5]. Previous studies on drug resistance show that ABC transporters are responsible for reducing the intracellular concentration of cytotoxic agents by increasing drug efflux [13]. MDR1 has been reported to mediate resistance to maytansinoids and antibody-maytansinoid conjugates [14,15]. Changes in isoforms or mutations and alterations in factors associated with microtubules might result in resistance to agents that bind to tubulin [16]. Besides, In patients receiving trastuzumab, resistance can be related to HER2 shedding, leading to a cleaved active form of HER2 [17]. Furthermore, the epitope recognized by trastuzumab can be hidden by molecules such as MUC4 [18]. Additionally, HER2 inhibition can be bypassed by an intrinsic activation of downstream pathways of HER2, for example, by PI3KCA mutation or loss of PTEN activity, or by activation of alternative pathways such as HER1/3 or IGF1R [19].

The resistance mechanisms to ADCs have yet to be well understood, as they are relatively new agents, but resistance to T-DM1 has been seen in pre-clinical and clinical reports [11,20,21]. Understanding the various resistance mechanisms will help design more effective ADCs and combination strategies to improve durable responses and survival [4]. In recent years, analysis of the gene networks using high-throughput approaches has been applied as promising tools with numerous clinical uses, including classification, detection, prognosis, and therapeutic response in cancer [[22], [23], [24]]. Using systems pharmacology and systems biology approaches offers new insights into the molecular understanding of drug resistance and helps detect gene signatures for precision medicine [25]. These systems-level insights can be applied to design more precise in silico models of biological circuits, resulting in cell and tumor responses [26,27].

This study uses bioinformatics analysis to investigate the genes involved in the molecular mechanism of EC drug resistance. It looks into the protein-protein interaction (PPI) networks, significant Gene Ontology (GO) terms and Kyoto Encyclopedia of Genes and Genomes (KEGG) pathways with a specific focus on possible gene hubs that have potential roles in the T-DM1 resistance in EC.

## 2 Methods

### 2.1 Bioinformatics workflow

**Fig. 1:**
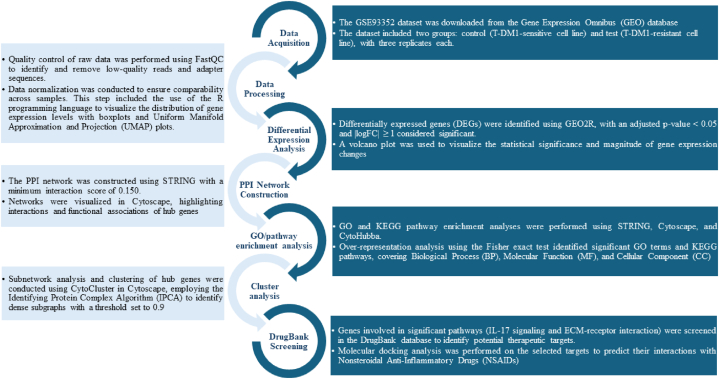
Bioinformatics workflow for data set filtering and transcriptomic analysis. This workflow outlines the steps from data acquisition and preprocessing to identifying potential therapeutic targets through molecular docking analysis.

A comprehensive bioinformatics workflow was implemented to process and analyze the data. This workflow included data acquisition, preprocessing, differential expression analysis, functional enrichment analysis, protein-protein interaction (PPI) network construction, cluster analysis, and DrugBank screening. Each step was meticulously performed to ensure accurate and reproducible results. A visual representation of the workflow is provided in Fig. 1.

### 2.2 Data source

The comprehensive screening of the Gene Expression Omnibus (GEO) database, encompassing 13 series of human esophageal cancer (EC), was conducted to identify relevant datasets for this study. The selection criteria were meticulously defined as follows: (1) studies focusing on resistance-related projects, (2) inclusion of *Homo sapiens* as the species, (3) data derived from expression profiling by microarray, (4) studies specifically related to esophageal cancer, (5) analyses involving esophageal cancerous and normal tissues, (6) datasets with at least two samples analyzed, and (7) availability of CEL files as a supplementary filter.

The dataset GSE93352 was chosen for detailed analysis following this thorough screening process. This dataset, deposited by Sauveur J et al., was obtained from the GEO database hosted by the National Center for Biotechnology Information (NCBI) (http://www.ncbi.nlm.nih.gov/geo/). The array data were generated using the platform GPL10558, specifically the Illumina HumanHT-12 V4.0 Expression BeadChip (Illumina, Inc.). The study design included two groups: a control group consisting of T-DM1-sensitive cell lines and a test group comprising T-DM1-resistant cell lines, each with three biological replicates.

### 2.3 Differentially expressed genes (DEGs) analysis

To identify differentially expressed genes in the GSE93352 dataset, we utilized the GEO2R online tool. This tool allows for comparing gene expression levels between samples, providing a straightforward and accessible method for DEG identification. We employed the Benjamini and Hochberg procedure to control the false discovery rate, ensuring the reliability of our findings. The analysis applied stringent filters, specifically using an adjusted p-value threshold of <0.05 and a minimum log fold change (|logFC|) of ≥1, to minimize the likelihood of false positives and focus on the most significant changes in gene expression. These criteria were selected to enhance the robustness and relevance of our results, providing a clear distinction between differentially expressed and non-differentially expressed genes.

### 2.4 Reconstruction of PPI networks and hub analysis

We employed the STRING database to analyze the protein-protein interactions among the up-regulated genes identified in this study (version 11.5, http://string-db.org) [28]. STRING provides a comprehensive resource of known and predicted PPIs, and for this analysis, we used a minimum interaction score of 0.150 to include both established and potentially novel interactions. The resulting PPI network was then visualized using Cytoscape (version 3.9.1), an open-source software platform for visualizing complex networks of biomolecular interactions [29].

We utilized the CytoHubba plugin (version 0.1) within Cytoscape. CytoHubba offers a user-friendly interface for identifying and ranking critical nodes (genes) within a network based on various computational algorithms [30]. Specifically, we used twelve algorithms: MCC, Degree, MNC, Betweenness, BottleNeck, Closeness, ClusteringCoefficient, DMNC, EcCentricity, EPC, Radiality, and Stress. The top four genes identified by each algorithm were considered hub genes, resulting in a final set of hub genes based on their prominence across these metrics. The interactions among these hub genes were then depicted in a subnetwork.

### 2.5 Gene Ontology and pathway enrichment analysis of the hub genes

Gene Ontology (GO) analysis is an essential tool for annotating genes and their associated products, providing valuable insights into the biological functions and processes they are involved in. This analysis helps elucidate significant biological features from high-throughput gene expression data, making it a critical component of our study [31]. Additionally, the Kyoto Encyclopedia of Genes and Genomes (KEGG) database was utilized to explore the broader functional contexts and utilities within biological systems, ranging from cellular processes to organismal interactions. KEGG's comprehensive data, derived from genome sequencing and other high-throughput technologies, offers a detailed understanding of the molecular mechanisms at play [32]. For our study, the enrichment analysis of the hub genes' subnetwork was conducted using GO and KEGG databases. We specifically focused on three GO categories: Molecular Function (MF), Cellular Component (CC), and Biological Process (BP). The analyses were performed using the STRING web-based application, allowing us to identify and characterize the fundamental biological pathways and molecular functions associated with our identified hub genes. This approach ensures a thorough understanding of the molecular and biological implications of these genes in the context of T-DM1 resistance in esophageal cancer, thus providing a solid foundation for further research and potential therapeutic strategies [28].

### 2.6 Cluster analysis of the network

CytoCluster (Version 2.1.0) was employed for the clustering of network nodes. We utilized the Identifying Protein Complex Algorithm (IPCA), a density-based algorithm that identifies dense subgraphs within protein interaction networks. IPCA operates by determining the weight of an edge through the calculation of shared neighbors between connected nodes and then calculates each node's weight by summing the weights of its incident edges [33]. A threshold of 0.9 was applied to ensure robustness in identifying meaningful clusters. The genes within each identified cluster were subsequently analyzed using STRING [34], to determine the KEGG pathways they are associated with. This methodological approach allows for a detailed exploration of functional interactions and pathway involvement among the clustered genes.

### 2.7 Statistical analysis

The statistical analysis employed GEO2R, using the Benjamini-Hochberg method to control the false discovery rate. Differential gene expression analysis considered genes with an adjusted p-value <0.05 and |logFC| ≥ 1 as significant. Data normalization and transformation were confirmed using boxplots and UMAP plots, ensuring comparability across samples. Gene Ontology (GO) and KEGG pathway enrichment analyses were performed using STRING, Cytoscape, and CytoHubba. Over-representation analysis with the Fisher exact test identified significant GO terms and KEGG pathways, covering Biological Process (BP), Molecular Function (MF), and Cellular Component (CC). Cluster analysis of protein-protein interaction (PPI) networks was conducted using CytoCluster in Cytoscape, employing the IPCA algorithm to identify dense subgraphs with a threshold set to 0.9.

### 2.8 Data processing

The GSE93352 dataset from GEO included T-DM1-sensitive and T-DM1-resistant groups, each with three replicates. Missing data were addressed using GEO2R's imputation methods. Normalization was verified through boxplot analyses, ensuring median-centered gene expression levels. Differentially expressed genes (DEGs) were identified with adjusted p-value <0.05 and |logFC| ≥ 1. Volcano plots visualized the statistical significance and magnitude of gene expression changes. PPI network analysis used STRING with a minimum interaction score of 0.150, and networks were visualized in Cytoscape, highlighting interactions and functional associations of hub genes.

### 2.9 Screening of genes in DrugBank

The results of the cluster analysis highlighted the importance of genes in cluster 2, particularly those involved in the IL-17 signaling pathway and the ECM-receptor interaction pathway. To explore potential therapeutic targets, we conducted a thorough search of these genes in the DrugBank database (https://go.drugbank.com/). This search aimed to identify approved drugs targeting the identified genes, providing a pathway to potential treatments. By focusing on these pathways and associated genes, we ensure that our findings are directly relevant to current therapeutic strategies and offer practical implications for clinical applications.

### 2.10 Receptor and ligand retrieval and preparation

The 3D structure of Prostaglandin G/H synthase 2 (PDB ID: 5ikr) protein was retrieved from the RCSB Protein Data Bank (https://www.rcsb.org/search). To prepare the structure for further analysis and docking simulations, all ligands and water molecules were removed, Kollman charges were added, and non-polar hydrogens were merged into the receptors. This process converted the structure into the PDBQT format suitable for subsequent computational studies. The chemical structures of the selected drugs were sourced from NCBI PubChem (https://pubchem.ncbi.nlm.nih.gov) and authorized. The structures were converted into the PDBQT file format using AutoDock tools, facilitating their use in docking simulations and ensuring compatibility with receptor analysis.

To ensure accurate structural representation of lymphoid enhancer-binding factor 1 and cadherin-11, whose tertiary structures were not available in existing protein databases, we utilized the online tool Robetta (https://robetta.bakerlab.org) for primary structure prediction with all-atom refinement [35]. The predicted 3D structures underwent rigorous evaluation using the MolProbity, ERRAT, and PROCHECK servers [36,37]. ERRAT analysis was employed to detect errors by assessing unbonded atom-atom interactions based on high-resolution structural statistics [38]. The PROCHECK-generated Ramachandran plot provided insight into the torsion angles of residues, highlighting whether they fall within allowed, preferred, or disallowed regions [39]. Furthermore, MolProbity analysis validated the quality of the predicted 3D structures of these macromolecules, including proteins, nucleic acids, and complexes [40]. This comprehensive validation process ensures that the predicted structures closely resemble naturally occurring protein structures, thereby enhancing the reliability of subsequent analyses.

### 2.11 Molecular docking

The interactions between the selected drugs and the receptors—prostaglandin G/H synthase 2, lymphoid enhancer-binding factor 1, and cadherin-11 receptors—were assessed using AutoDock Vina. AutoDock Vina is recognized for its accuracy in scoring ligand-receptor interactions, particularly for ligands of typical biological size. It accounts for spherically symmetric hydrogen bonding potentials, implicit hydrogens, and hydrophobicity, making it well-suited for this type of molecular docking study [41]. The docking simulations provided valuable insights into the binding affinities and potential efficacy of the selected drugs against these target receptors, thereby enhancing the reliability of subsequent analyses. The docking results were analyzed using PyMOL version 1.1.7 and Discovery Studio version 4.5 [42]. The best conformations for each ligand-receptor complex were selected based on two critical criteria: the maximum number of bonds between the ligands and receptors and the lowest docked binding energy. These criteria ensured the identification of the most stable and biologically relevant interactions, providing insights into the potential efficacy of the selected drugs.

## 3 Results

### 3.1 Screening of differentially expressed genes (DEGs)

**Fig. 2:**
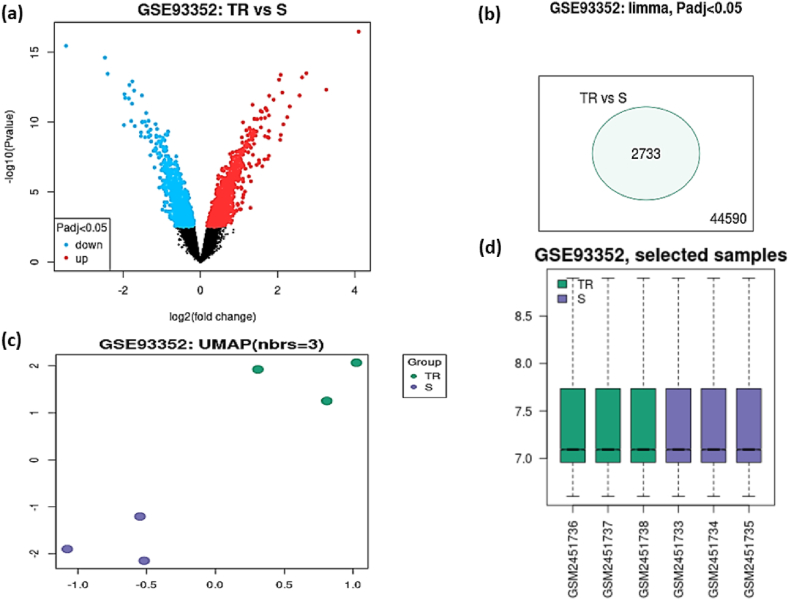
Overview of DEGs between T-DM1-sensitive and T-DM1-resistant esophageal cell lines. (a) Volcano plot showing DEGs identified with significant expression changes (red: up-regulated; blue: down-regulated; black: no difference). (b) Venn diagram illustrating the initial set of 2733 DEGs identified using an adjusted p-value <0.05. (c) UMAP plot depicting the separation of T-DM1-sensitive and T-DM1-resistant cell lines. (d) Boxplot displaying normalized expression values across the samples (Y-axis: normalized expression levels).

The GSE93352 dataset was downloaded from the GEO database and validated with GEO2R before analysis. The study included two groups: control (T-DM1-sensitive cell line) and test (T-DM1-resistant cell line), each with three replicates. A volcano plot (Fig. 2a) displays statistical significance (−log10 p-value) versus magnitude of change (log2 fold change) in gene expression. In the initial filtering stage, a total of 2733 genes were identified as differentially expressed using an adjusted p-value <0.05 (Fig. 2b). The Uniform Manifold Approximation and Projection (UMAP) plot (Fig. 2c) shows clear separation between the groups, indicating distinct expression profiles. Data normalization was confirmed using an R-generated boxplot (Fig. 2d), demonstrating that the dataset is median-centered and cross-comparable, suitable for further analysis using GEO2R.

To refine our analysis and focus on the most significant gene expression changes, we applied stricter criteria of adjusted p-value <0.05 and |logFC| ≥ 1, identifying 157 DEGs, including 107 up-regulated and 50 down-regulated genes. Further analysis was conducted on the up-regulated genes to identify potential drug targets.

### 3.2 Reconstruction of PPI network and hub analysis

**Fig. 3:**
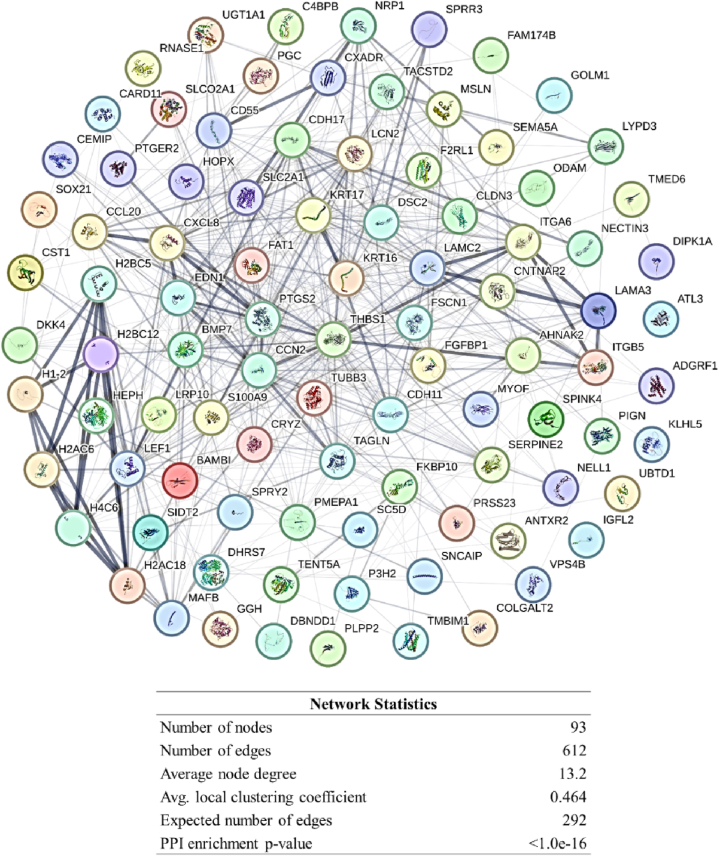
Network of up-regulated genes in T-DM1 resistant EC cell lines, including their known interacting partners, visualized using Cytoscape software.

The analysis of up-regulated genes using STRING and Cytoscape revealed a comprehensive PPI network, as shown in Fig. 3. The hub analysis, conducted using the CytoHubba plugin, identified nine key genes (Table 1). These genes represent critical nodes in the PPI network, indicating their potential importance in the biological processes underlying T-DM1 resistance. The specific subnetwork consisting of these hub genes and their interactions is illustrated in Fig. 4.

**Table 1:** List of hub genes identified using CytoHubba. These genes were determined to be the most connected and influential within the PPI network based on the analysis of various ranking algorithms provided by the software.

#	Node	Gene ID	Ensemble	Gene Description	Rank	Ranking Method	Other names	Location
1	CTGF	1490	ENSG00000118523	cellular communication network factor 2	1, 2, 2	MCC, MNC, Degree	CCN2; NOV2; HCS24; IGFBP8	6q23.2
2	CDH17	1015	ENSG00000079112	cadherin 17	2,	MCC	HPT1; CDH16; HPT-1	8q22.1
3	THBS1	7057	ENSG00000137801	thrombospondin 1	3, 1, 1	MCC, MNC, Degree	TSP; THBS; TSP1; TSP-1; THBS-1	15q14
4	CXCL8	3576	ENSG00000169429	C-X-C motif chemokine ligand 8	4, 4, 4	MCC, MNC, Degree	IL8; NAF; GCP1; LECT; LUCT; NAP1; GCP-1; LYNAP; MDNCF; MONAP; NAP-1; SCYB8	4q13.3
5	NRP1	8829	ENSG00000099250	neuropilin 1	1	DMNC	NP1; NRP; BDCA4; CD304; VEGF165R	10p11.22
6	ITGB5	3693	ENSG00000082781	integrin subunit beta 5	2	DMNC	integrin, beta 5	3q21.2
7	EDN1	1906	ENSG00000078401	endothelin 1	3	DMNC	ET1; QME; PPET1; ARCND3; HDLCQ7	6p24.1
8	FAT1	2195	ENSG00000083857	FAT atypical cadherin 1	4	DMNC	FAT; ME5; CDHF7; CDHR8; hFat1	4q35.2
9	PTGS2	5743	ENSG00000073756	prostaglandin-endoperoxide synthase 2	3, 4	MNC, Degree	COX2; COX-2; PHS-2; PGG/HS; PGHS-2; hCox-2; GRIPGHS	1q31.1

**Fig. 4:**
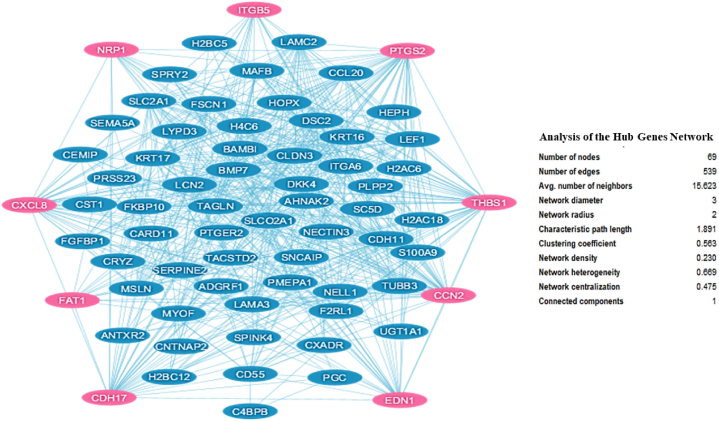
PPI network of hub genes among the up-regulated genes in T-DM1 resistant EC cell lines, constructed using the CytoHubba plugin. The network highlights the central role of these hub genes within the broader interaction network.

### 3.3 Gene Ontology and pathway enrichment analysis of hub genes

**Fig. 5:**
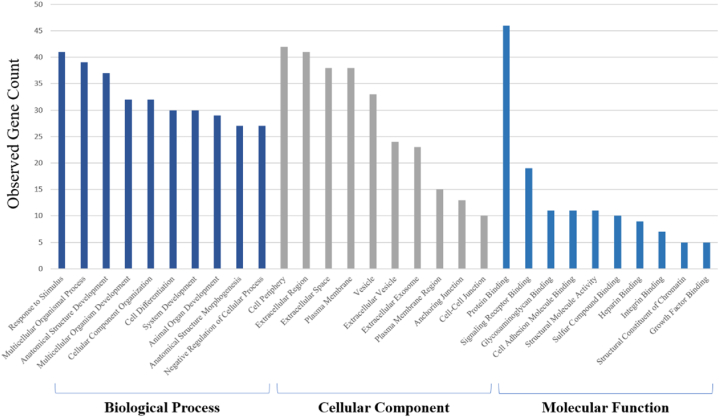
Gene Ontology (GO) enrichment analysis for Biological Process (BP), Cellular Component (CC), and Molecular Function categories (MF). The bar chart represents the number of observed genes associated with each GO term. The x-axis shows the GO terms, grouped into BP, CC, and MF). The y-axis indicates the count of observed genes. The blue bars correspond to BP (dark blue) and MF (light blue) categories, while the gray bars represent CC categories. This figure provides a comprehensive overview of the enriched GO terms for the differentially expressed genes in the study.

**Fig. 6:**
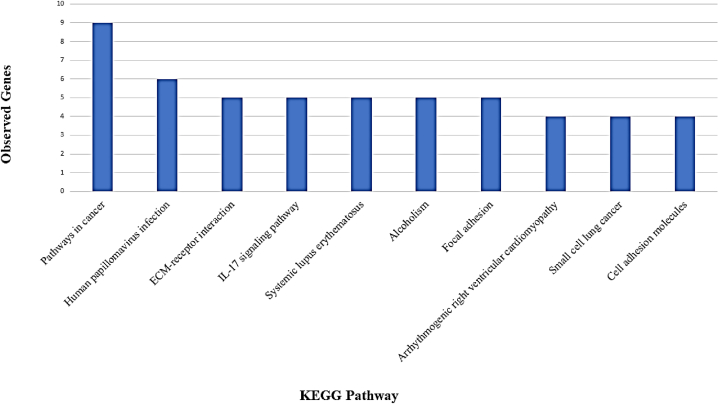
Kyoto Encyclopedia of Genes and Genomes (KEGG) pathway analysis of the determined subnetwork of hub genes in T-DM1 resistant EC cell line using STRING version 10.

Gene Ontology (GO) analysis is a widely recognized method for annotating genes and gene products, providing insights into the biological aspects of high-throughput genome or transcriptome data. This includes categorizing molecular functions, cellular components, and biological processes [43]. The results of GO analysis and pathway enrichment are depicted in Fig. 5, Fig. 6**.**

The GO enrichment analysis revealed significant associations of differentially expressed genes with specific biological processes, cellular components, and molecular functions. In the BP category, the most enriched processes included “Response to stimulus” (41 genes), “Multicellular organismal process” (39 genes), and “Anatomical structure development” (37 genes). These processes highlight the complex involvement of the differentially expressed genes in the physiological responses and developmental mechanisms of esophageal carcinoma cells. For the CC category, the top enriched components were “Cell periphery” (42 genes), “Extracellular region” (41 genes), and “Extracellular space” (38 genes). This suggests a significant role of these genes in cellular localization and the extracellular environment, which may influence cell-cell interactions and the tumor microenvironment. The MF category analysis indicated a predominant enrichment in “Protein binding” (46 genes), followed by “Signaling receptor binding” (19 genes) and “Glycosaminoglycan binding” (11 genes). These functions are crucial for signal transduction, molecular interactions, and cellular communication, which are often altered in cancerous cells. This comprehensive GO analysis provides a detailed understanding of the functional implications of the identified differentially expressed genes, suggesting their potential roles in tumor biology and resistance mechanisms in esophageal carcinoma.

Based on KEGG enrichment analysis, the prominent pathways are significantly enriched in Pathways in cancer, Human papillomavirus infection, ECM-receptor interaction, IL-17 signaling pathway, Systemic lupus erythematosus, Alcoholism, Focal adhesion, Arrhythmogenic right ventricular cardiomyopathy, Small cell lung cancer, and Cell adhesion molecules. These pathways are illustrated in Fig. 6.

### 3.4 Cluster analysis of the network

**Fig. 7:**
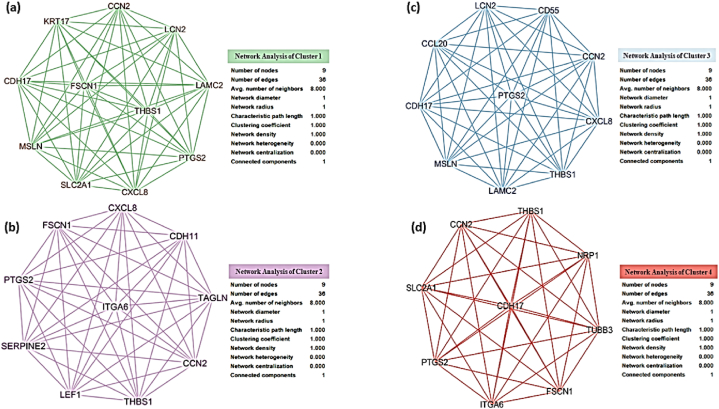
Clusters of hub genes and their associated networks in T-DM1 resistant EC cell lines. (a) Cluster 1, (b) Cluster 2, (c) Cluster 3, (d) Cluster 4. Each cluster represents a group of interconnected genes identified through network analysis. The analysis includes the number of nodes, edges, and various network properties, such as the clustering coefficient and network density.

The cluster analysis of the network of hub genes identified in T-DM1-resistant EC cell lines revealed four significant clusters. Fig. 7 depicts the visual representation of these clusters, highlighting the network structure and properties of each cluster.

Cluster 1 contains 11 nodes and 55 edges, with pathways enriched in cancer-related processes, including the IL-17 signaling pathway, microRNAs in cancer, and Human papillomavirus infection. The nodes in this cluster exhibit high connectivity, as evidenced by the clustering coefficient of 1.000 and a network density of 1.000. Cluster 2 includes ten nodes and 45 edges associated with pathways such as the Kaposi sarcoma-associated herpesvirus infection, NF-kappa B signaling pathway, ECM-receptor interaction, and Small cell lung cancer. This cluster also shows a high level of interconnectivity, with network properties similar to Cluster 1. Cluster 3 and Cluster 4 both contain nine nodes each, with 36 and 45 edges, respectively. These clusters are involved in pathways related to microRNAs in cancer, Malaria, Bladder cancer, and other biological processes. The network properties of these clusters, such as the clustering coefficient and network heterogeneity, indicate distinct subnetwork characteristics that may play crucial roles in T-DM1 resistance.

The pathway enrichment analysis for these clusters, as summarized in Table 2, reveals a range of biological processes and pathways that may contribute to resistance mechanisms in these EC cell lines. These pathways include significant ones, such as the IL-17 signaling pathway and ECM-receptor interaction, highlighting potential targets for further investigation and therapeutic intervention.

**Table 2:** Pathways enriched in the top four clusters of hub genes in T-DM1 resistant EC cell lines. Each cluster's rank, number of nodes, edges, and associated KEGG pathways are listed.

Cluster Rank	Nodes	Edges	Pathways
1	11	55	Pathways in cancer
			IL-17 signaling pathway
			MicroRNAs in cancer
			Human papillomavirus infection
			Bladder cancer
			Malaria
2	10	45	Pathways in cancer
			Kaposi sarcoma-associated herpesvirus infection
			MicroRNAs in cancer
			Human papillomavirus infection
			Malaria
			Bladder cancer
			Arrhythmogenic right ventricular cardiomyopathy
			NF-kappa B signaling pathway
			ECM-receptor interaction
			IL-17 signaling pathway
			Small cell lung cancer
3	10	45	IL-17 signaling pathway
			Human papillomavirus infection
			Malaria
			Bladder cancer
4	9	36	MicroRNAs in cancer

### 3.5 Screening of genes in DrugBank

The pathway enrichment analysis identified the IL-17 signaling pathway and ECM-receptor interaction pathway as pivotal in T-DM1 resistance. Further cluster analysis corroborated the importance of these pathways. Consequently, genes involved in these pathways, including CXCL8, FSCN1, PTGS2, SERPINE2, LEF1, THBS1, CCN2, TAGLN, CDH11, and ITGA6, were selected for a detailed search in DrugBank (https://go.drugbank.com/) to identify potential therapeutic targets. The search results, summarized in Table 3, reveal that among these genes, only PTGS2, LEF1, and CDH11 have approved drugs that target them. Notably, a variety of drugs target PTGS2 and CDH11; however, Nonsteroidal Anti-Inflammatory Drugs (NSAIDs) emerged as a common class of drugs targeting both genes. This finding highlights the potential therapeutic relevance of NSAIDs in addressing T-DM1 resistance associated with these pathways.

**Table 3:** List the approved drugs targeting the genes involved in the IL-17 signaling pathway and ECM-receptor interaction pathway.

Hub gene	Drug name	Drug group	Drug category	Drug targets	Drug background	*P*athway
*PTGS2*	Naproxen	approved, vet-approved	Anti-Inflammatory Agents, Non-Steroidal	Prostaglandin G/H synthase 1, Prostaglandin G/H synthase 2, Pepto-streptococcal albumin-binding protein	The NSAID naproxen, approved initially in 1976, is an effective, well-tolerated first-line analgesic for acute and rheumatic pain with multiple formulations, including combination therapies.	Arachidonic acid metabolism
*PTGS2*	Diclofenac	approved, vet-approved	Anti-Inflammatory Agents, Non-Steroidal	Prostaglandin G/H synthase 2, Prostaglandin G/H synthase 1	Diclofenac, an NSAID designed based on other anti-inflammatory drugs, is a first-line therapy for acute and chronic pain and inflammation, though often combined with misoprostol to prevent gastric ulcers.	Arachidonic acid metabolism
*PTGS2*	Mefenamic acid	approved	Anti-Inflammatory Agents, Non-Steroidal	Prostaglandin G/H synthase 2, Prostaglandin G/H synthase 1	Diclofenac is an NSAID that inhibits cyclooxygenase to exert analgesic, anti-inflammatory, and antipyretic pharmacological effects.	Arachidonic acid metabolism
*PTGS2*	Etoricoxib	approved, investigational	Anti-Inflammatory Agents, Non-Steroidal	Prostaglandin G/H synthase 2	Etoricoxib, a COX-2 selective NSAID approved in over 60 countries, treats rheumatoid arthritis, osteoarthritis, back pain, and other inflammatory conditions by inhibiting prostaglandin synthesis from arachidonic acid.	Arachidonic acid metabolism
*PTGS2, CDH11*	Celecoxib	approved, investigational	Anti-Inflammatory Agents, Non-Steroidal	Prostaglandin G/H synthase 2, Carbonic anhydrase 2, Carbonic anhydrase 3, Cadherin-11, 3-phosphoinositide-dependent protein kinase 1, Sialidase-1	Selective cyclooxygenase-2 inhibitors like celecoxib reduce gastrointestinal bleeding risk compared to other nonsteroidal anti-inflammatory drugs, making them useful for managing arthritis symptoms. As preliminary clinical trials suggest, these agents may also have chemo-preventive and therapeutic efficacy against cancers.	Arachidonic acid metabolism
*LEF1*	Etacrynic acid	approved, investigational	Acetates	Lymphoid enhancer-binding factor 1, Sodium/potassium-transporting ATPase subunit alpha-1, Solute carrier family 12 member 1, Glutathione S-transferase P	Loop diuretics inhibit electrolyte symporters in the nephron, causing enhanced urinary excretion of sodium, potassium, and chloride, increased urine output, and decreased extracellular fluid volume.	–

### 3.6 Receptor preparation

To ensure the selected 3D structures are ideal and consistent with native protein conformations, the predicted structures were validated using ERRAT, MolProbity, and Ramachandran plot analyses. The MolProbity score, clash score, Ramachandran plot evaluation, and ERRAT results confirmed the predicted structures (Table 4). This table shows the evaluation of the tertiary structure of the receptors, specifically Lymphoid enhancer-binding factor 1 and Cadherin-11, using several validation metrics: ERRAT, MolProbity score, Clashscore, and Ramachandran plot analysis. These metrics are essential for assessing the quality and accuracy of the predicted 3D structures, which are crucial for subsequent molecular docking and functional analysis.

**Table 4:** Evaluation of the tertiary structure of receptors using ERRAT, MolProbity, and Ramachandran plot servers.

Predicted structure	ERRAT	MolProbity score	Clashscore	Residues in allowed region (%)	Residues in disallowed region (%)
Lymphoid enhancer-binding factor 1	90.1786	1.18 (99 % of the most ideal structural resolution)	1.47 (99 % of the most ideal structural resolution)	99.4 %	0.6
Cadherin-11	90.1809	0.87 (100 % of the most ideal structural resolution)	1.07 (99 % of the most ideal structural resolution)	96	0.4

The ERRAT score provides an overall quality assessment of the protein structure based on non-bonded atomic interactions. An ERRAT score above 90 % is generally considered indicative of a high-quality model. In our analysis, lymphoid enhancer-binding factor 1 achieved an ERRAT score of 90.1786, indicating a good model quality. Cadherin-11 also showed a high ERRAT score of 90.1809, suggesting reliable structural prediction.

The MolProbity score combines various validation checks, including clash scores and geometry analyses, to comprehensively assess model quality. A lower score indicates a better model. Both receptors scored well on this metric: Lymphoid enhancer-binding factor 1 had a MolProbity score of 1.18, representing 99 % of the most ideal structural resolution. Cadherin-11 had an even lower score of 0.87, indicating that the model falls within 100 % of the most ideal structural resolution.

The Clashscore measures the number of steric clashes per 1000 atoms, where a lower score is better. It assesses the physical plausibility of the model by checking for atomic overlaps: Lymphoid enhancer-binding factor 1 had a Clashscore of 1.47, reflecting minimal atomic clashes and a physically plausible model. Cadherin-11 had a Clashscore of 1.07, further supporting the quality of the predicted structure.

The Ramachandran plot assesses the stereochemistry of protein structures by plotting the phi (φ) and psi (ψ) angles of amino acid residues. It helps evaluating the conformational angles of the residues: for Lymphoid enhancer-binding factor 1, 99.4 % of residues were in the allowed regions, with only 0.6 % in the disallowed regions. This high percentage in the allowed region indicates a well-modeled structure. For Cadherin-11, 96 % of residues were in the allowed regions, with a very low 0.4 % in the disallowed regions, further indicating a reliable structure.

The results in Table 4 demonstrate that both protein models, Lymphoid enhancer-binding factor 1 and Cadherin-11, exhibit high-quality structural characteristics. These metrics collectively confirm the reliability and accuracy of the predicted 3D structures, which are critical for the subsequent stages of molecular docking and functional studies. The high ERRAT scores, low MolProbity scores, favorable Clashscores, and excellent Ramachandran plot statistics all point to well-resolved and reliable models.

### 3.7 Binding interactions of selected drugs with receptors

The interactions between the selected drugs and receptors are detailed in Table 5. All selected drugs exhibited a suitable docking score with their respective receptors. The complexes Lymphoid enhancer-binding factor 1 with ethacrynic acid (binding energy: −5.6 kcal/mol) and Cadherin11 with celecoxib (binding energy: −6.7 kcal/mol) showed relatively lower binding affinities at the predicted active sites of the receptors. In contrast, other drug-receptor complexes demonstrated a higher binding affinity. The 2D and 3D structural visualizations of these interactions are also presented in Table 5.

**Table 5:** Summary of the docking interactions of the selected drugs against the receptors.

Compound	Docking interaction		Binding Energy (kcal/mol)
Prostaglandin G/H synthase 2-Naproxen			−8.3
Prostaglandin G/H synthase 2-Diclofenac			−8.4
Prostaglandin G/H synthase 2-Mefenamic acid			−8.6
Prostaglandin G/H synthase 2-Etoricoxib			−7.5
Lymphoid enhancer-binding factor 1- Etacrynic acid			−5.6
Prostaglandin G/H synthase 2 - Celecoxib			−7.8
Cadherin11-Celecoxib			−6.7

## 4 Discussion

This comprehensive bioinformatics analysis of TDM-1-resistant esophageal cancer (EC) cell lines has provided crucial insights into the molecular mechanisms of resistance and potential therapeutic targets. We identified nine key hub genes (CTGF, CDH17, THBS1, CXCL8, NRP1, ITGB5, EDN1, FAT1, and PTGS2) associated with TDM-1 resistance. These genes are involved in critical biological processes and pathways contributing to resistance mechanisms.

CTGF was identified as a significant driver of TDM-1 resistance, influencing various mechanisms, including pro-survival signaling, EMT induction, and extracellular matrix (ECM) remodeling [44]. Targeting CTGF could potentially reverse resistance by disrupting these pathways [45]. CXCL8 plays a substantial role in promoting resistance through angiogenesis, immune suppression, and drug metabolism alteration. Inhibiting CXCL8 signaling could be an effective strategy to restore drug sensitivity [[46], [47], [48]]. Integrin β5 (ITGB5) contributes to resistance by enhancing cell proliferation, reducing apoptosis, and maintaining stem-like traits. It is linked with key pathways like focal adhesion and the PI3K-Akt signaling pathway, suggesting a role in broader drug resistance beyond TDM-1 [[49], [50], [51], [52]].

FAT1, an atypical cadherin, is implicated in resistance through mechanisms such as EMT induction and Wnt/β-catenin signaling activation. Its role in promoting stemness and immune suppression makes it a critical target for overcoming resistance [5,53,54].

Prostaglandin-endoperoxide synthase 2 (PTGS2), also known as cyclooxygenase-2 (COX-2), mediates resistance by fostering an immunosuppressive microenvironment [55], promoting angiogenesis [55,56], and supporting cancer stem cell populations [57]. Its inhibition could enhance the efficacy of TDM-1 by mitigating these effects [5,58].

While the primary focus has been on the nine hub genes, CDH11 and LEF1 emerged as notable genes within the ECM-receptor interaction and IL-17 signaling pathways, respectively [59]. CDH11 is a cell adhesion molecule involved in the epithelial-mesenchymal transition (EMT), a critical process in cancer metastasis and drug resistance. CDH11 has been identified as a potential therapeutic target with existing drugs available, highlighting its clinical relevance in managing T-DM1-resistant esophageal cancer [60]. LEF1, a transcription factor within the Wnt signaling pathway, is associated with cell proliferation, differentiation, and migration. Its role in cancer biology, particularly in promoting tumor growth and metastasis, makes it a valuable target for therapeutic intervention. The availability of agents targeting components of the Wnt pathway further underscores the importance of including LEF1 in our discussion of potential therapeutic strategies [61]. These genes were prioritized due to their translational potential, emphasizing the importance of exploring approved drugs and developing new therapies to improve treatment outcomes for patients with resistant cancer phenotypes. In the context of TDM-1 resistance in esophageal cancer, the identified hub genes are associated with significant GO terms that highlight their involvement in crucial biological processes, molecular functions, and cellular components. These GO terms provide insights into the broader functional roles of these genes and their contributions to resistance mechanisms. The hub genes are involved in key biological processes such as cell adhesion, angiogenesis, apoptotic signaling regulation, and immune response modulation. These processes are critical in cancer progression, particularly in facilitating metastasis, immune evasion, and resistance to apoptosis. In addition, the hub genes are linked to functions such as protein binding, receptor binding, and enzyme regulation. These molecular functions underscore the role of the hub genes in mediating interactions within the cellular environment, influencing signaling pathways, and modulating enzymatic activities that can affect drug metabolism and efficacy. The analysis also indicates a significant presence of these genes in extracellular regions, cell membranes, and vesicles. This localization is essential for understanding how these genes participate in cell-cell communication, interaction with the extracellular matrix, and intracellular trafficking processes that can impact drug delivery and resistance. These GO terms collectively illustrate the multifaceted roles of the identified hub genes in TDM-1 resistance, providing a comprehensive view of the underlying molecular mechanisms. By targeting these processes and functions, new therapeutic strategies can be developed to overcome resistance and improve treatment outcomes for EC patients.

Functional enrichment analysis revealed significant involvement of the identified genes in pathways such as “Pathways in cancer,” “ECM-receptor interaction,” and “IL-17 signaling.” These pathways play essential roles in cancer progression and resistance [50,62], offering potential targets for therapeutic intervention. The persistent significance of the IL-17 signaling pathway suggests it as a central mechanism in TDM-1 resistance [63].

The DrugBank analysis highlighted the potential role of NSAIDs in targeting PTGS2, which is implicated in resistance mechanisms. NSAIDs like Diclofenac, Mefenamic acid, Celecoxib, Naproxen, and Etoricoxib showed high binding affinity to PTGS2, indicating their potential to suppress TDM-1-resistant cancer cells by modulating inflammation and immune responses [64,65].

The findings from this study provide a detailed understanding of the molecular underpinnings of TDM-1 resistance in EC. By highlighting key genes and pathways, including potential drug targets like CDH11 and LEF1, this research opens avenues for developing new therapeutic strategies. Further investigation into these pathways and the potential repurposing of NSAIDs could lead to improved outcomes for patients with resistant esophageal cancer.

## 5 Conclusion

This comprehensive bioinformatics analysis deeply explains the molecular mechanisms governing TDM-1 resistance in esophageal cancer. The identification of nine hub genes (CTGF, CDH17, THBS1, CXCL8, NRP1, ITGB5, EDN1, FAT1, and PTGS2) and their associated pathways offers potential targets for therapeutic interventions. For instance, CTGF has been shown to influence drug resistance through mechanisms such as extracellular matrix remodeling and cytoprotective autophagy. CXCL8 promotes resistance by facilitating angiogenesis and altering drug metabolism. Integrin β5 (ITGB5) is implicated in resistance by enhancing cell proliferation and survival signaling. Enriching these hub genes in pathways such as IL-17 signaling and ECM-receptor interaction further underscores their significance in TDM-1 resistance. Moreover, the potential role of Nonsteroidal Anti-Inflammatory Drugs (NSAIDs) in mitigating T-DM1 resistance presents an intriguing avenue for further investigation, as docking results revealed that NSAIDs such as Diclofenac and Celecoxib exhibit significant binding affinity to PTGS2, a key mediator in resistance mechanisms. This research contributes significantly to the field and lays the foundation for future studies to improve treatment outcomes for EC patients.

## Ethics statement

This study does not require informed consent.

## Consent for publication

Not applicable.

## Funding

This article had no funding support.

## Data availability statement

The data associated with this study has not been deposited into a publicly available repository. This is because the data is included in the article, supplemental material, or referenced within the article.

## CRediT authorship contribution statement

**Fateme Yazdani:** Writing – original draft, Methodology, Investigation, Formal analysis. **Negar Mottaghi-Dastjerdi:** Writing – review & editing, Writing – original draft, Visualization, Validation, Supervision, Project administration, Methodology, Investigation, Formal analysis, Data curation, Conceptualization. **Behzad Shahbazi:** Writing – original draft, Methodology, Formal analysis, Data curation. **Khadijeh Ahmadi:** Writing – original draft, Methodology, Formal analysis, Data curation. **Abozar Ghorbani:** Writing – review & editing, Methodology. **Mohammad Soltany-Rezaee-Rad:** Writing – review & editing, Project administration, Formal analysis. **Hamed Montazeri:** Writing – review & editing, Methodology. **Farzane Khoshdel:** Writing – review & editing, Data curation. **Pietro Hiram Guzzi:** Writing – review & editing, Formal analysis.

## Declaration of generative AI and AI-assisted technologies in the writing process

During the preparation of this work, the authors utilized Grammarly and ChatGPT for grammar checking, language editing, and improving readability. After using these tools/services, the authors reviewed and edited the content as needed and take full responsibility for the content of the publication.

## Declaration of competing interest

The authors declare that they have no known competing financial interests or personal relationships that could have appeared to influence the work reported in this paper.
